# Effect of *Lepidium meyenii* Walp. on Semen Parameters and Serum Hormone Levels in Healthy Adult Men: A Double-Blind, Randomized, Placebo-Controlled Pilot Study

**DOI:** 10.1155/2015/324369

**Published:** 2015-09-01

**Authors:** Ingrid Melnikovova, Tomas Fait, Michaela Kolarova, Eloy C. Fernandez, Luigi Milella

**Affiliations:** ^1^Department of Crop Sciences and Agroforestry, Faculty of Tropical AgriSciences, Czech University of Life Sciences Prague, Kamycka 129, 165 21 Prague 6, Czech Republic; ^2^Department of Gynecology and Obstetrics, 1st Medical Faculty, Charles University, Apolinarska 18, 128 51 Prague 2, Czech Republic; ^3^Department of Agroecology and Biometeorology, Faculty of Agrobiology, Food and Natural Resources, Czech University of Life Sciences Prague, Kamycka 129, 165 21 Prague 6, Czech Republic; ^4^Department of Science, University of Basilicata, Viale dell'Ateneo Lucano 10, 85100 Potenza, Italy

## Abstract

*Background/Aims. *Products of *Lepidium meyenii* Walp. (maca) are touted worldwide as an alimentary supplement to enhance fertility and restore hormonal balance. Enhancing properties of maca on semen parameters in animals were previously reported by various authors, but we present to the best of our knowledge the first double-blind, randomized, placebo-controlled pilot trial in men. The aim of this study was to evaluate the effects of maca on semen parameters and serum hormone levels in healthy adult men.* Methods. *A group of 20 volunteers aged 20–40 years was supplied by milled hypocotyl of maca or placebo (1.75 g/day) for 12 weeks. Negative controls of semen were compared to the samples after 6 and 12 weeks of maca administration; negative blood controls were compared to the samples after 12 weeks of treatment.* Results. *Sperm concentration and motility showed rising trends compared to placebo even though levels of hormones did not change significantly after 12 weeks of trial.* Conclusion. *Our results indicate that maca possesses fertility enhancing properties in men. As long as men prefer to use alimentary supplement to enhance fertility rather than prescribed medication or any medical intervention, it is worth continuing to assess its possible benefits.

## 1. Introduction

The infertility caused by impaired spermatogenesis and semen parameters is a public health problem which has become an issue of great concern. As long as current therapeutic management is often associated with side effects and no significant efficacy [1], need of an alternative treatment arises. For this purpose, plants with an ethnopharmacological reputation have been widely investigated for the identification of their possible biological activities [2–5] or for the treatment of several ailments [6–8].* Lepidium meyenii* Walp. from the Brassicaceae family may be applied. This Andean bulbous crop called maca has been used for centuries by native inhabitants to boost overall vitality and treat infertility in humans and domestic animals [9]. Even today, maca products attract widespread interest for its claimed fertility enhancing properties in both male and female. In fact it was recently demonstrated that maca root may alleviate SSRI-induced sexual dysfunction in postmenopausal women [8]. Maca was reported to enhance sexual desire [10, 11] and improved mild erectile dysfunction [12] in men. Its semen quality improving properties have been reported by various studies in animals: mice [13], bulls [14], and rams [15]; but convincing scientific evidence for its efficacy on semen in men is still lacking. According to the observed biological activities of its lipid fraction [16], studies regarding maca have focused on a group of unique nonpolar, long-chain fatty acid* n*-benzylamides called macamides, which are considered as chemotaxonomic markers used to assess its quality [17]. Nevertheless, the mechanism of its enhancing properties has not yet been fully discovered.

Twenty apparently healthy men were treated with maca or placebo for 12 weeks in order to evaluate its effect on semen parameters and serum hormone levels. In particular we have monitored five reproductive hormones (luteinizing hormone, follicle-stimulating hormone, prolactin, estradiol, and testosterone), two thyroid hormones (free thyroxin and thyroid-stimulating hormone), and semen parameters (total sperm count, sperm concentration, morphology, and motile sperm count) to evidence the effects of maca supplement on the mentioned parameters. As recommended by previous studies [17–19] macamides content was quantified by High-Pressure Liquid Chromatography with UV Detector (HPLC-UV).

## 2. Material and Methods

### 2.1. Subjects

Twenty healthy men of 20–40 years were included in the study. They were nonsmokers and at least 3 months before and during the study did not use hormonal treatment, anabolics, or any medical substances which could change their serum hormone levels. All the participants signed agreements to be involved in the study after being informed of its purpose, possible benefits, and risks and approval from an independent Ethics Committee was obtained.

Using a prospective, randomized, placebo-controlled, double-blind design [20], 20 patients were randomly assigned into the trial group (11 patients) and the control group (9 patients). Group assignment for all subjects was determined using a random table prior to initiation of the study. The sequence of assignments was unknown to any of the investigators. Each assignment was kept in a sealed envelope, and the order in numeric number was shown on the outside of the envelope. Thus, the orders could not be changed. Envelopes were arranged in order. The principal investigator generated this random selection a few months before recruiting the first subject. No significant differences in age, physical condition, and clinical stage of disease between the two groups were found.

### 2.2. Maca and Placebo

Gelatinized and powdered maca was provided by the Peruvian company Andean Roots SRL and delivered to the Czech Republic in 2012. The plant material, yellow type of maca, was harvested in the Cerro de Pasco region of the central Peruvian Andes at the altitude between 4200 and 4500 meters above sea level. During the gelatinization, original dried hypocotyls of maca were rehydrated and exposed to short-term elevated pressure under moist conditions. This standard process of sample preparation decomposed the starch component and increased the digestibility of the product; after gelatinization the sample was dried again to less than 7% humidity. The content of the six most abundant macamides, the quality markers of maca, was analyzed in gelatinized sample by HPLC-UV following the methodology previously described [18, 19]. A placebo was selected on the basis of color and taste similarity to maca powder; milled apple fiber (Country Life) and sucrose (Cukrovar Vrbatky a.s.) were used in the ratio 3 : 2.

### 2.3. Design of Experiment

To evaluate the effect of maca, this pilot study was designed as a 12-week, double-blind, placebo-controlled, randomized, parallel trial in which active treatment by gelatinized maca was compared to a placebo. Twenty men were randomly divided as previously described. Powder was dosed into the gelatin enterosolvent capsules, each of which contained 350 mg; the daily dose was five capsules, equal to 1.75 g. Semen samples were collected before, in the 6th and in the 12th week of the treatment. Samples of blood were collected before and after the trial.

### 2.4. Assessment of Semen Parameters

The samples of semen were obtained by masturbation after 3 days of sexual abstinence and evaluated in the Assisted Reproduction Center Apolinar, Department of Obstetrics and Gynecology, 1st Faculty of Medicine, Charles University, and General Faculty Hospital in Prague. Seminal analysis was performed according to the guidelines of the World Health Organization [21].

### 2.5. Hormone Assay

Blood samples were collected on the same day as the semen samples. Five reproductive hormones (luteinizing hormone, follicle-stimulating hormone, prolactin, estradiol, and testosterone) and two thyroid hormones (free thyroxin and thyroid-stimulating hormone) were measured by routine immune-analytical methods in the Central Laboratory of General Faculty Hospital, 1st Faculty of Medicine, Charles University in Prague.

### 2.6. Statistical Analysis

All the data were analyzed using Statistica 10 software. As homogeneity of variance assumptions were not satisfied in all cases, a nonparametric Mann-Whitney *U* test was used to analyze intergroup differences and a Kruskal-Wallis *H* test was performed for analyzing data of more than two groups. A *P* value is based on the Chi-square distribution and a *P* value of less than 0.05 was considered statistically significant.

## 3. Results

From 20 volunteers, two men appeared to suffer from oligozoospermia; therefore, they had to be excluded from the trial. Out of 18 remaining volunteers, 7 consumed placebo and 11 maca.

### 3.1. Semen Parameters

**Figure 1:**
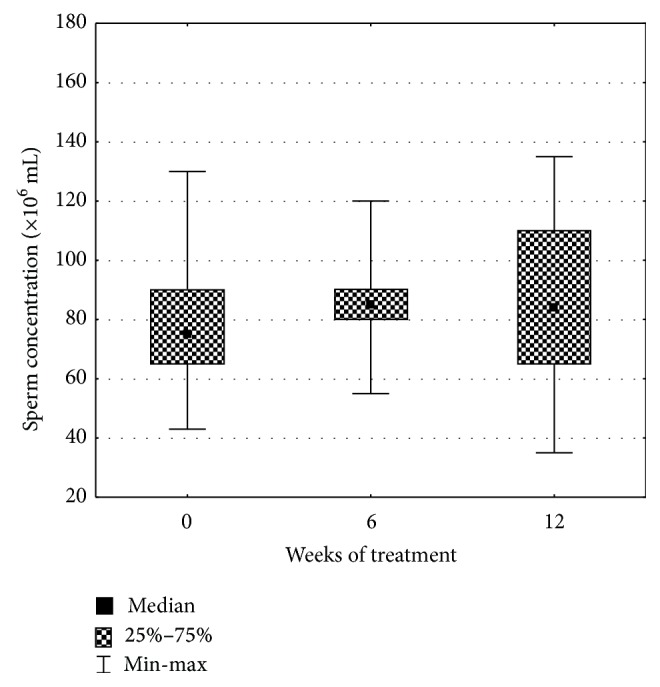
Influence of maca treatment on sperm concentration.

**Figure 2:**
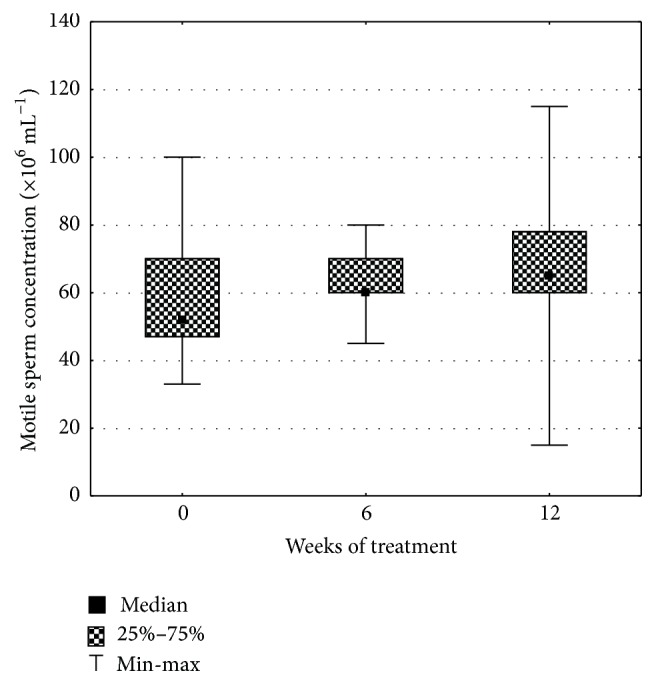
Influence of maca treatment on motile sperm concentration.

Not any statistically significant differences between semen parameters in different collection dates as well as maca versus placebo groups were found. This could be due to higher within groups variation. We found, however, that all assessed quality parameters showed rising trends in the maca group after 12 weeks of the trial (Table 1). Total sperm count increased by 20%, sperm concentration by 14%, motile sperm count by 14%, progressively motile sperm count by 18%, semen volume by 9%, and normal morphology of sperms by 21% (Figures 1 and 2). In the placebo group, total sperm count increased also by 20%, but sperm concentration did not change and motile sperm count and normal sperm morphology decreased by 10% and 14%, respectively.

### 3.2. Serum Hormone Levels

Statistically significant difference in level of prolactin between the baseline of maca and placebo group was found; therefore, prolactin was not included in the hormonal analysis. No substantial changes of other hormone levels were observed in the blood serum after 12 weeks of maca or placebo administration (Table 2).

### 3.3. Macamides Content

The content of six main abundant macamides in maca powder is shown in Table 3. Among them* n*-benzylhexadecanamide,* n*-benzyl-(9Z.12Z)-octadecadienamide, and* n*-benzyl-(9Z.12Z.15Z)-octadecatrienamide were the most abundant; the content has been found to be 1.68 ± 0.29, 1.02 ± 0.17, and 1.02 ± 0.16 mg/g of dried weight of plant material (mg/g DW), respectively.

## 4. Discussion

We wish to evaluate maca's semen quality enhancing properties by, to the best of our knowledge, the first double-blind, randomized, placebo-controlled trial in healthy men and to contribute to the current scientific evidence of this crop with a pronounced ethnopharmacological reputation. This pilot study appears to extend the knowledge of maca's properties and supports several findings of previous studies. Our results showed rising trends of semen parameters after 12 weeks of maca administration and correlate with two previously conducted studies in men, which lacked any control group [22, 23]. Subjects consumed 3 g of powdered maca in both above mentioned studies. Gonzales et al. [22] reported in nine healthy volunteers a statistically significant increase of sperm concentration by 35%, total sperm count by 84%, and count of motile sperm even by 109% after 16 weeks of treatment. Tancara et al. [23] studied ten patients with clinically diagnosed infertility. Authors did not find any improvement in sperm concentration or total sperm count but reported a statistically significant increase of motile sperm count by 10% and of normal sperm morphology by 12% after 12 weeks of maca consumption; these findings are consistent with ours.

Maca's semen enhancing properties have also been described in several scientifically rigorous studies in animals [13–15]. Gonzales et al. [13] reported increased stages of spermiation and mitosis of germ cells during the spermatogenic cycle in male rats, demonstrating also that yellow maca and black maca are the varieties responsible to increase sperm count and sperm motility whereas red maca had no effect. Rubio et al. [24] indicated that maca also reversed lead acetate induced damage of spermatogenesis in male rats. These findings suggest that maca improves sperm formation. This is in line with an increased motile sperm count and count of sperms with normal morphology in our and the above named studies in men.

In order to demonstrate whether maca influences fertility on a hormonal basis, levels of selected hormones in blood serum were measured. We did not find any substantial changes in comparison to the baseline or a control group. These results reinforce previous studies in rats [25] and humans [26] and* in vitro* [27], which did not show an influence of maca consumption on hormone levels. On the contrary, Oshima et al. [28] reported increased level of progesterone in female and testosterone in male mice and Uchiyama et al. [21] described significant elevation of luteinizing hormones in female mice with a daily dose of 3–30 g maca powder per kg. However, maca forms part of native Andean inhabitants' diet; daily doses used in animal models seem to be unfeasible for humans.

The mechanism of the fertility enhancing properties of maca remains unconfirmed, but some aphrodisiac activities have been related to its lipidic fraction [13], which contains mainly fatty acids and macamides. Macamides are used to assess the quality of maca products; therefore, we quantified the most abundant of them. Their content was comparable to the already published average values obtained by the analysis of samples from different locations in Peru. The amount of the most common macamide,* n*-benzylhexadecanamide, was determined as 1.68 mg/g, while in previous studies these values differed between 1.39–3.68 mg/g [19] and 0.49–4.57 mg/g [29]. The content of macamides varies significantly; therefore, we encourage the recommendations of previous studies to determine their content before each experiment.

From the viewpoint of trial design, the daily doses and the length of treatment would be expected to play a crucial role. Values in spermiograms of our volunteers showed rising trends between 6th week and 12th week of treatment. Thus, the fact that Gonzales et al. [22] obtained more significant results after 16 weeks of trial supports the hypothesis that the length of maca administration influences semen parameters positively. On the other hand his team described no significant impact if the daily dose of maca was reduced from 3 to 1.5 g.

Hirsh [30] declares that subfertility affects one in 20 men. This correlates well with our results since two volunteers of our group suffered from oligozoospermia. These results support evidence that subfertility affects a considerable amount of young men. Nevertheless, sperm parameters achieved by 18 men in this study were higher than recent WHO reference values for human semen characteristics [31].

Maca is touted as a potent over-the-counter (OTC) supplement. As long as men prefer to use OTC products to enhance fertility rather than prescribed medication or any medical intervention, it is worth continuing to assess its possible benefits [1]. Our results indicate that maca possesses some of its claimed properties. Hence, we decided to continue maca oriented research in order to evaluate its potential. A trial considering the effect of maca in patients suffering oligozoospermia is currently being developed.

## Figures and Tables

**Table 1:** Mean semen parameter values during maca and placebo treatment (mean ± standard error; Kruskal-Wallis *H* test).

		*n*	Normal sperm morphology (%)	Sperm concentration (×10^6^ mL^−1^)	Progressively motile sperm count (×10^6^ mL^−1^)	Motile sperm count (×10^6^ mL^−1^)	Total sperm count (×10^6^)	Semen volume (mL)
Maca	0 weeks	11	18.18 ± 1.68	77.27 ± 6.79	47.72 ± 4.33	58.00 ± 5.40	277.90 ± 31.18	3.66 ± 0.31
	6 weeks	11	22.22 ± 2.22	86.11 ± 7.48	51.66 ± 2.88	63.22 ± 3.47	241.11 ± 25.54	2.92 ± 0.33
	12 weeks	11	22.00 ± 2.00	87.80 ± 9.86	56.20 ± 7.19	66.30 ± 8.36	332.31 ± 58.98	3.99 ± 0.31
	*P* value		*0.29*	*0.52*	*0.28*	*0.39*	*0.42*	*0.11*
	*H*	*3.25*	*1.31*	*2.56*	*1.89*	*1.75*	*4.46*

Placebo	0 weeks	7	20.71 ± 1.70	106.71 ± 15.59	70.00 ± 12.53	83.42 ± 13.73	295.56 ± 69.580	2.77 ± 0.53
	6 weeks	7	23.33 ± 2.10	100.00 ± 10.24	60.00 ± 8.560	71.66 ± 8.620	395.16 ± 152.57	3.57 ± 1.01
	12 weeks	7	17.85 ± 3.75	100.28 ± 12.04	65.71 ± 11.09	74.85 ± 11.43	355.28 ± 96.940	3.44 ± 0.64
	*P* value		*0.44*	*0.83*	*0.89*	*0.72*	*0.87*	*0.73*
	*H*	*0.23*	*0.38*	*0.23*	*0.67*	*0.27*	*0.63*

**Table 2:** Baseline and posttreatment serum level of hormones in maca and placebo-treated subjects (mean ± standard error; Mann-Whitney *U* test).

		*n*	LH^*∗*^	FSH^*∗∗*^	Estradiol	Testosterone	fT4^*∗∗∗*^	TSH^*∗∗∗∗*^
			(IU L^−1^)	(IU L^−1^)	(nmol L^−1^)	(nmol L^−1^)	(pmol L^−1^)	(mIU L^−1^)
Maca	0 weeks	11	4.03 ± 0.50	4.40 ± 0.37	0.10 ± 0.01	19.92 ± 1.75	16.31 ± 0.29	2.35 ± 0.30
	12 weeks	11	2.90 ± 0.33	3.75 ± 0.37	0.10 ± 0.01	20.10 ± 2.24	16.91 ± 0.62	1.58 ± 0.23
	*P* value	*0.09*	*0.23*	*0.76*	*0.88*	*0.95*	*0.07*

Placebo	0 weeks	7	3.82 ± 0.50	4.46 ± 0.50	0.09 ± 0.01	19.43 ± 2.25	14.02 ± 0.68	2.92 ± 1.63
	12 weeks	7	2.99 ± 0.57	3.55 ± 0.64	0.11 ± 0.01	18.63 ± 2.15	15.28 ± 0.72	2.63 ± 1.38
	*P* value	*0.23*	*0.23*	*0.17*	*1.00*	*0.23*	*1.00*

^*∗*^Luteinizing hormone; ^*∗∗*^follicle-stimulating hormone; ^*∗∗∗*^free thyroxin; ^*∗∗∗∗*^thyroid-stimulating hormone.

**Table 3:** Content of the six most abundant macamides in maca powder (mg/g DW).

Macamide	Mean ± SE
Methoxy-*n*-benzyl-(9Z.12Z.15Z)-octadecatrienamide	0.12 ± 0.05
*n*-Benzyl-(9Z.12Z.15Z)-octadecatrienamide	1.02 ± 0.17
Methoxy-*n*-benzyl-(9Z.12Z)-octadecadienamide	0.10 ± 0.01
*n*-Benzyl-(9Z.12Z)-octadecadienamide	1.02 ± 0.16
*n*-Benzylhexadecanamide	1.68 ± 0.29
*n*-Benzyl-(9Z)-octadecanamide	0.41 ± 0.08
